# Exploring the associations between cardiovascular health measured with the CANHEART model and early cognitive impairment in a middle-aged population in Korea

**DOI:** 10.4178/epih.e2021044

**Published:** 2021-07-13

**Authors:** Ye Jin Jeon, Ji Heon Lee, Hyeon Chang Kim, Sun Jae Jung

**Affiliations:** 1 Department of Public Health, Yonsei University Graduate School, Seoul, Korea; 2 Yonsei University College of Medicine, Seoul, Korea; 3 Department of Preventive Medicine, Yonsei University College of Medicine, Seoul, Korea

**Keywords:** Cardiovascular diseases, Cognitive dysfunction, Inflammation, Korea

## Abstract

**OBJECTIVES:**

Both cardiovascular health (CVH) and inflammation are associated with cognition, and inflammation is also associated with CVH. However, limited information has been reported on these factors in the Korean population. The objective of our study was to investigate the influence of inflammation on the association between CVH and cognition using a cross-sectional design.

**METHODS:**

Data were obtained from the Cardiovascular and Metabolic Diseases Etiology Research Center baseline study. Participants who completed fasting serum analysis, questionnaires, and cognitive function tests were included in the analysis, whereas those with a history of autoimmune disease were excluded. The CVH in Ambulatory Care Research Team health index metrics, including smoking, physical activity, healthy diet, obesity, history of hypertension, and diabetes, were used to assess CVH. Cognitive function was evaluated with the Korean version of the Mini-Mental State Estimation for Dementia Screening. Inflammatory status was assessed based on a high-sensitivity C-reactive protein (hs-CRP) test.

**RESULTS:**

Among 2,622 total participants (mean age, 57.2 years; 1,792 women), 13%, 58%, and 29% had poor, intermediate, and ideal CVH, respectively. Logistic regression analysis demonstrated that CVH was significantly associated with cognitive function only in women. A stratified analysis showed that cognitive impairment due to CVH was not associated with hs-CRP levels. When the same analyses were conducted for each CVH component, the only component affecting the association was hypertension history in men.

**CONCLUSIONS:**

CVH is not significantly associated with cognitive decline in the middle-aged Korean population. Inflammation did not play a significant modifying role in this relationship.

## INTRODUCTION

Degenerative dementia has become a serious health problem in Korea, as the prevalence of dementia is almost 10.0% in the population above the age of 65 years. The management of dementia in Korea costs approximately 20 million Korean won (KRW) per person annually in 2015 [1]. Moreover, dementia imposes a psychosocial burden on both patients and caregivers [2]. Thus, more effective risk evaluation and management for dementia could alleviate economic, psychological, and social problems [3]. For example, the early diagnosis of dementia reduces the cost of care for Alzheimer disease (AD) by US$100 billion annually in the United States [3].

The current treatments for AD are rivastigmine, donepezil, galantamine, and memantine [4]. It has been revealed that an early diagnosis and treatment of AD improves the prognosis [3,5]. Unfortunately, the clinical diagnosis of AD is being made by positron emission tomography and cerebrospinal fluid studies, which are expensive or invasive, and only give information after AD has developed to some extent [4]. To start treatment of AD earlier, it is necessary to identify a biomarker that reflects the risk of AD [6].

Many previous studies have suggested that poor cardiovascular health (CVH) is significantly associated with low cognitive function and dementia [7,8]. A previous study conducted in France showed that higher CVH scores were associated with a lower risk of dementia and cognitive decline [8]. Additionally, levels of inflammatory markers such as high-sensitivity C-reactive protein (hs-CRP) are related to both CVH and cognitive function [9,10]. The Centers for Disease Control and Prevention and the American Heart Association have already proposed hs-CRP as a useful tool for determining cardiovascular disease risk [10]. C-reactive protein (CRP) could predict cardiovascular risk as much as lipid levels do [11]. A report has also stated that individuals who have high physical activity and consume a healthy diet show both low inflammatory levels and high CVH [12]. Inflammatory status is also associated with cognitive function [13]. CRP could therefore be a useful marker of memory and visuospatial impairment in elderly individuals [14]. The plasma level of CRP was found to be higher in patients with AD than in healthy controls [15]. Higher hs-CRP levels were also related to reduced cerebral microstructural integrity [16]. Consequently, inflammation could be considered a possible factor connecting CVH and cognitive impairment.

Previously, CVH scores were usually measured by the American Heart Association (AHA) Life’s Simple 7 tool [17]. However, no significant association was found between CVH and early cognitive dysfunction in a Korean cohort based on the AHA Life’s Simple 7 tool [18]. The CANHEART model was developed to evaluate CVH in the Canadian population by the Canadian Community Health Survey, inspired by the AHA Life’s Simple 7 tool. It successfully predicted CVH risk in Canadian population. Furthermore, unlike the AHA Life’s Simple 7 tool, the CANHEART health index does not require laboratory testing, meaning that it is highly convenient and accessible for evaluating patients [19].

Therefore, we aimed to investigate the association between CVH and cognitive decline, and then to explore cognitive decline according to hs-CRP levels and CVH to determine whether hs-CRP is valuable as a biomarker for early cognitive dysfunction.

## MATERIALS AND METHODS

### Study population

The Cardiovascular and Metabolic Diseases Etiology Research Center (CMERC) study was conducted between 2013 and 2018 to investigate risk factors for cardiovascular and metabolic disease. The CMERC study enrolled participants meeting the following criteria: (1) aged 30 years to 64 years, (2) having an urban residence in Seoul or nearby, (3) able to articulate their own opinions, (4) no history of overt cardiovascular diseases (lifetime), or malignant cancer (within the previous 2 years), (5) no concurrent enrollment in other clinical trials, and (6) not pregnant at baseline. The details of the study are described elsewhere [20]. Cognitive function at age 50 years or older (n=2,663) was evaluated with the Mini-Mental State Examination (MMSE), which was administered to the entire cohort. Forty-one participants with lifetime autoimmune disease or with incomplete socio-demographic information, including education, household income, or marital status, were excluded. Finally, a total of 2,622 participants were included in this analysis.

### Assessment of cardiovascular health and the cardiovascular risk score

We used the AHA Life’s Simple 7 tool (2010) and the Cardiovascular Health in Ambulatory Care Research Team (CANHEART) health index (2013) [19]. In the AHA Life’s Simple 7 tool, each of the 7 components was categorized as “poor,” “intermediate,” or “ideal” according to the AHA’s guidelines; details of the criteria applied to each component are described in Supplementary Material 1. The CANHEART health index was developed to evaluate the Canadian population and references data from the Canadian Community Health Survey, a cross-sectional telephone survey of self-reported health status, determinants, and health service use by Canadian adults aged 20 years or older. Unlike the AHA Life’s Simple 7 tool, the CANHEART health index does not require a laboratory test. The CANHEART health index includes health behaviors such as smoking, physical activity, healthy diet, obesity, diabetes mellitus, and hypertension history [19]. We scored the sum of each component in both CVH indices, and all were equally weighted (AHA Life’s Simple 7 tool score: 0-7; CANHEART health index score: 0-6).

### Measurement of cognitive function

Cognitive function was tested with the Korean version of the Mini Mental State Examination for Dementia Screening (MMSE-DS), administered by trained interviewers to participants aged at least 50 years. The MMSE-DS measures cognitive function using questions to evaluate various categories of cognitive function, including time and place, orientation, registration, attention and calculation, memory recall, speaking, and visual construction. If any item had a missing value, the total MMSE-DS score was discarded (n=6). According to a previous validation study in Korea, the test showed excellent internal consistency and diagnostic accuracy (area under the curve, 0.895 [0.880 to 0.911]) [21]. In this study, individuals with an MMSE-DS score below 24 were categorized as having low cognitive function.

### Measurement of the high-sensitivity C-reactive protein level as an indicator of inflammatory status

Inflammatory status was assessed using 8-hour fasting morning blood plasma samples. Plasma levels of hs-CRP were analyzed with a turbid immunoassay (ADVIA1800 Auto Analyzer; Siemens Medical Solutions, Malvern, PA, USA). According to the manufacturer, the detection range for the hs-CRP assay is 0.01 mg/L to 1,000 mg/L, with a sensitivity of 0.2 mg/L (men: median, 0.64; interquartile range, 0.40 to 1.36; women: median, 0.60; interquartile range, 0.34 to 1.18).

### Covariates

During the baseline assessment, the participants were asked questions regarding demographic characteristics, socioeconomic status, medical/medication history, family history, and lifestyle factors (smoking, drinking, sleeping, physical activity, and food consumption) by a trained interviewer using a general questionnaire with a standardized protocol. Household income was classified into quartiles (< 24.0, 24.0 to < 34.6, 34.6 to < 49.0, and ≥ 49.0 million KRW/yr). Education level was categorized into groups according to the educational curriculum in Korea (elementary school or below, middle school, high school, college, or above). Marital status was classified as “never married,” “widowed,” “separated/divorced,” or “married and living together.” Smoking and drinking were categorized as “never,” “past,” or “current.” Physical activity was assessed with a Korean version of the International Physical Activity Questionnaire-short form, which enquires about the frequency of each of the following activities: walking, moderate-intensity activity, and vigorous activity. The validity of the Korean version of the International Physical Activity Questionnaire-short form has been confirmed, but it has low reliability [22].

In this study, blood samples were collected after an 8-hour fast and transported on the registration date to the institution where the analysis was performed. Total cholesterol, high-density lipoprotein cholesterol, low-density lipoprotein cholesterol, and triglyceride levels were measured enzymatically (ADVIA1800 Auto Analyzer, Siemens Medical Solutions). Fasting blood glucose levels were measured with a colorimetric assay (ADVIA1800 Auto Analyzer, Siemens Medical Solutions).

Height was measured to the nearest 0.1 cm with an optical linear encoder scale, and body weight was measured to the nearest 0.1 kg on a digital scale. Body mass index (BMI) was calculated as weight divided by height squared (kg/m^2^). Upper arm blood pressure was measured 3 times after the participant had been seated and at rest for at least 5 minutes. We utilized the average of the second and third blood pressure measurements.

### Statistical analysis

The chi-square test and analysis of variance (F-test) were used to compare baseline differences in covariates of the MMSE-DS score categories (cut-off=24). Continuous variables are shown as mean and standard deviation, while categorical variables are shown as frequency and percentage. A logistic regression model examined the association between the CVH level and cognitive dysfunction. The final model was adjusted for age [23], educational level [24], household income level [25], marital status [26], current drinking status [27], mean systolic blood pressure [28], total cholesterol [29], and fasting glucose level [30]. The selection of confounders was based on previous studies, and in the variance inflation factor (VIF) test for the detection of multicollinearity, all covariates included in the model satisfied the criterion (VIF< 10). Stratified analyses by hs-CRP level were conducted with the same covariates, and we used tertiles to classify the hs-CRP levels. Sensitivity analyses were conducted for comparison with pre-imported data using the same logistic regression model (n=2,241), excluding participants who did not complete the dietary questionnaire. All statistical analyses were performed using SAS version 9.4 (SAS Institute Inc., Cary, NC, USA).

### Ethics statement

All participants provided written informed consent, and the study protocol was approved by the Yonsei University College of Medicine Hospital Institutional Review Board (4-2013-0661). All procedures complied with the ethical standards of the relevant national and institutional committees on human experimentation as per the Helsinki Declaration of 1975 (revised in 2008).

## RESULTS

The characteristics of the study participants (n=2,622) are shown in Table 1. Men and women constituted 31.7% and 68.3% of the study group, respectively. The mean age of participants was 57.2 years. Participants were classified by cognitive function as determined by the MMSE-DS score. Based on the MMSE-DS score, 2,535 participants were classified as having normal cognitive function and 87 (3.3%) participants exhibited low cognitive function (MMSE-DS score < 24). Lower cognitive function scores were associated with older age, women, low education level, low family income, non-smoking, and poor CVH (p<0.05).

### CANHEART health index and cognitive function

Overall, poor CVH (evaluated by the CANHEART health index; poor=0-1, intermediate=2-5, and ideal=6) was significantly associated with low cognitive function (evaluated by the MMSE-DS, score < 24) after full adjustment (odds ratio [OR], 1.99; 95% confidence interval [CI], 1.01 to 3.92) (Table 2). We additionally checked linearity with the same model, but it was not significant (p_trend_=0.143). The association between CVH and cognitive decline in men is presented in Table 3; compared to ideal CVH, poor CVH was associated with low cognitive function, with an OR of 9.33, although this association was not significant. The linear association between CVH and low cognitive function was significant (p_trend_=0.037). In the subgroup analysis by hs-CRP tertiles, only the lower tertile showed a significant association between CVH and cognitive function, and the effect size was strong (hs-CRP lower tertile: OR, 3.46; 95% CI, 1.14 to 10.45). In the stratified analysis conducted across hs-CRP tertiles, the risk of cognitive decline was highest in the group with high hs-CRP levels and poor CVH, although this relationship was not statistically significant. The associations between CVH and cognitive decline in women are presented in Table 3. Compared to the ideal CANHEART health index, poor CVH was associated with low cognitive function in women, with an OR of 2.03 (95% CI, 1.00 to 4.11).

The risk of cognitive decline was highest in those with an intermediate hs-CRP level and poor CVH, but this relationship was not significant. In a previous analysis conducted in the same population, we assessed the association between the AHA Life’s Simple 7 score (poor, 0-2; intermediate, 3-4; ideal; 5-7) and cognitive decline. Poor CVH measured by the AHA Life’s Simple 7 score was positively associated with low cognitive function compared to ideal CVH, but this relationship was not statistically significant in either men or women (Supplementary Material 2; men: OR; 1.71; 95% CI, 0.37 to 7.93; women: OR, 1.20; 95% CI, 0.55 to 2.58). For sensitivity analyses, we conducted the same analyses in preimputed data for dietary intake (n=2,241); the characteristics of included and excluded participants are presented in Supplementary Material 3. Overall, the association between poor CVH and low cognitive function was strong in the lower hs-CRP tertile (Supplementary Material 4).

### Components of the CANHEART health index and cognitive function

The associations of each component of CANHEART health index, including current smoking status, overweight status, leisure physical activity, fruit and vegetable consumption, hypertension, and diabetes, with cognitive decline in men are presented in Table 4. Low cognitive function was only associated with hypertension in men, with an OR of 3.79 (95% CI, 1.29 to 11.10). A definite association between hypertension and low cognitive function was represented in those with high hs-CRP levels, with an OR of 7.68 (95% CI, 1.66 to 35.59). In women, there was no significant association between the CANHEART health index components and low cognitive function (Table 4).

## DISCUSSION

The association between CVH and early cognitive function was not significant, and the level of hs-CRP did not affect the risk of cognitive dysfunction. The CVH assessment results obtained using the CANHEART health index did not show an association with cognitive function in either men or women, except in women with poor CVH scores. A stratified analysis across hs-CRP tertiles revealed no mediatory effect in the relationship between CVH and cognitive function. Although the presence of hypertension in men was associated with cognitive dysfunction, no other component of CVH had a significant association with cognition in either men or women.

Many previous studies have suggested that poor CVH is associated with cognitive decline. According to prospective cohort studies conducted in the United Kingdom, dementia incidence among participants with a low CVH level evaluated by the AHA Life’s Simple 7 tool was approximately twice as high as that of normal participants. Inflammation levels have also been speculated to play a role in modifying the association between CVH and cognitive function. Low-grade chronic inflammation can increase the risk of atherosclerosis and insulin resistance, which are the leading mechanisms in the development of cardiovascular disease [31]. Combined inflammation was significantly associated with memory and psychomotor speed. Moreover, chronic inflammation results in an increase in cytokine levels, causing hypersecretion of cortisol. Increased steroid levels reduce the synthesis of neurotrophic factors and prevent the repair of damaged neuronal networks. Furthermore, data from a cohort study of Japanese-American men showed that high levels of hs-CRP increased the risk of all types of dementia [14].

Our study presented different results from those of previous studies. We found that CVH levels did not show an association with early cognitive decline in our study population. The current study also confirmed that inflammation levels had no significant association with cognitive dysfunction. Several explanations for these differences could be suggested. Since the study population was only in an early stage of cognitive impairment, not the stage of dementia, the influence of CVH and inflammatory status on cognitive decline could be weak [32]. Since the criterion in previous studies was dementia, the results of those studies could be different from ours [7,8]. This possibility is supported by some findings suggesting that CRP levels could be different in different stages of disease [33]. In addition, variations across different cohorts could provide an explanation. Reportedly, the fruit and vegetable consumption in Korea is about 377.0 g/d [34], whereas that in France is 342 g/d and that in the United Kingdom is 258 g/d [35]. Since a comparatively high consumption of fruits and vegetables could contribute to a delay in cognitive decline, the effects of CVH and hs-CRP could be masked [36]. It is also possible that unknown differences in Eastern/Western population health-related factors might have an effect. Differences in the methods of measuring cognitive dysfunction could be another reason for inconsistent study outcomes. In our study, the Korean version of the MMSE-DS was used, whereas previous studies used a combination of cognitive tests [7]. The differences between the AHA’s Life’s Simple 7 tool and the CANHEART model might also cause dissimilar results. The AHA’s Life’s Simple 7 tool includes smoking, physical activity, healthy diet, BMI, total cholesterol, blood pressure, and fasting plasma glucose [19]. In comparison, the CANHEART index includes smoking, leisure physical activity, fruit and vegetable consumption, BMI, hypertension, and diabetes; several of these components are subjective and may be easily affected by recall bias [37]. The total cholesterol level was also excluded from CANHEART components. Due to this difference in CVH measurements, our findings may differ from those of previous studies. To compare the CANHEART health index and the AHA Life’s Simple 7 tool, we estimated the association between the AHA Life’s Simple 7 tool and cognitive function; as shown in Supplementary Material 2, the confidence interval for the ORs was wider with the CANHEART health index.

According to our knowledge, this is the first investigation to assess the association between the CANHEART health index and cognitive function in Korean population; as such, our results are meaningful. However, the current study had several limitations. First, there was a small number of participants with low cognitive function. In this study, only 87 of the 2,622 participants had an MMSE-DS score below 24. The smaller sample size than previous studies may compromise the statistical power of the current study, as the post-hoc power of the current analysis was found to be about 55.6% (n=2,622). A previous research study conducted in France included 745 dementia patients in a total study population of 6,626 [8]. Another study conducted in the United Kingdom included 347 dementia patients in a total population of 7,899 [7]. Second, the CANHEART model used for measuring CVH was originally designed by Canadian researchers, meaning that its validity is unknown for the Korean population. Third, our study had a cross-sectional design; therefore, the temporal association between CVH and cognitive function could not be verified. Fourth, the elements that may have caused conflicting results in studies investigating associations between CVH and cognitive function in different populations are unknown. Several factors such as genetic differences between races [38], the degree of fruit and vegetable consumption [39,40], or environmental and cultural influences [41] could be relevant. Further investigations to develop an appropriate index for the Korean population are needed.

Our study found that poor CVH levels showed a non-significant association with early cognitive decline, and inflammation levels did not have a modifying effect on this relationship. Several recommendations can be made for designing future studies to ascertain why our results conflicted with those of previous studies. A larger study population would be important, since in our study, the small size of the study group may have been the reason for non-significance. The measurement of other peripheral inflammatory factors, such as interleukin-6 or tumor necrosis factor-alpha, could be recommended instead of hs-CRP. Comparing early cognitive decline with late stages of cognitive decline, such as AD, would also be needed in further studies.

## Figures and Tables

**Table 1.** Descriptive characteristics of study participants

Characteristics			Total (n=2,622)	Cognitive function		p-value
				Normal, MMSE DS≥24 (n=2,535)	Low, MMSE DS<24 (n=87)	
Age (yr)			57.2±3.9	57.2±3.9	58.3±3.9	0.010
Gender						
	Men		830 (31.7)	818 (32.3)	12 (13.8)	<0.001
	Women		1,792 (68.3)	1,717 (67.7)	75 (86.2)	
Educational attainment (yr)						
	≤6		218 (8.3)	192 (7.6)	26 (29.9)	<0.001
	6-9		326 (12.4)	295 (11.6)	31 (35.6)	
	9-12		1,118 (42.6)	1,091 (43.0)	27 (31.0)	
	>12		960 (36.6)	957 (37.8)	3 (3.5)	
Family income (annual)						
	Q1		653 (24.9)	612 (24.1)	41 (47.1)	<0.001
	Q2		772 (29.4)	743 (29.3)	29 (33.3)	
	Q3		510 (19.5)	502 (19.8)	8 (9.2)	
	Q4		687 (26.2)	678 (26.8)	9 (10.3)	
Marital status						
	Unmarried		27 (1.0)	27 (1.1)	0 (0.0)	0.330
	Married					
		Death of spouse	151 (5.8)	145 (5.7)	6 (6.9)	
		Separated	146 (5.6)	138 (5.4)	8 (9.2)	
		Living together	2,298 (87.6)	2,225 (87.8)	73 (83.9)	
Smoking						
	Non-smoker		1,909 (72.8)	1,835 (72.4)	74 (85.1)	0.020
	Former smoker		481 (18.3)	470 (18.5)	11 (12.6)	
	Current smoker		232 (8.9)	230 (9.1)	2 (2.3)	
Drinking						
	Non-drinker		670 (25.6)	645 (25.4)	25 (28.7)	0.460
	Former drinker		126 (4.8)	124 (4.9)	2 (2.3)	
	Current drinker		1,826 (69.6)	1,766 (69.7)	60 (69.0)	
Regular physical activity^1^						
	No		705 (26.9)	682 (26.9)	23 (26.4)	0.920
	Yes		1,917 (73.1)	1,853 (73.1)	64 (73.6)	
Body mass index			24.0±2.9	24.0±2.9	24.3±2.6	0.360
Ever had hypertension^2^						
	No		2,018 (77.0)	1,955 (77.1)	63 (72.4)	0.310
	Yes		604 (23.0)	580 (22.9)	24 (27.6)	
SBP (mmHg)			120.3±15.1	120.4±15.2	119.2±14.3	0.480
DBP (mmHg)			76.6±9.6	76.6±9.6	74.7±8.3	0.060
Ever had DM2						
	No		2,438 (93.0)	2,359 (93.1)	79 (90.8)	0.420
	Yes		184 (7.0)	176 (6.9)	8 (9.2)	
Fasting insulin (μIU/mL)			8.8±3.7	8.8±3.7	8.7±3.2	0.670
Fasting glucose (mg/dL)			93.9±20.3	93.9±20.5	93.0±14.4	0.580
HbA1c (%)			5.8±0.7	5.8±0.7	5.8±0.5	0.580
hs-CRP (mg/L)			1.5±3.7	1.4±3.6	1.8±4.2	0.430
CANHEART health index^3^						
	Poor		342 (13.0)	323 (12.7)	19 (21.8)	0.040
	Intermediate		1,520 (58.0)	1,477 (58.3)	43 (49.4)	
	Ideal		760 (29.0)	735 (29.0)	25 (28.7)	

Values are presented as mean±standard deviation or number (%). MMSE-DS, Mini-Mental State Examination-Dementia Screening; SBP, systolic blood pressure; DBP, diastolic blood pressure; DM, diabetes mellitus; HbA1c, hemoglobin A1c; hs-CRP: high-sensitivity C-reactive protein.

1 Participants walking at least 30 minutes per day were grouped into the regular physical activity group.

2 Self-reported disease history.

3 CANHEART health index and its criteria defined by the Cardiovascular Health in Ambulatory Care Research Team in 2014.

**Table 2.** Associations between the CANHEART health index and cognitive function by hs-CRP tertiles

CANHEART health index^1^			Low cognitive function (MMSE-DS<24)^2^			p for trend
			Age and sex-adjusted model	Model 1, additionally adjusted for SES and drinking status	Model 2, additionally adjusted for health status	
Total participants (n=2,622)						0.143
	Poor (n=342)		2.38 (1.28-4.42)	1.62 (0.86-3.03)	1.99 (1.01-3.92)	
	Intermediate (n=1,520)		0.95 (0.58-1.57)	0.76 (0.46-1.26)	0.83 (0.50-1.38)	
	Ideal (n=760)		1.00 (reference)	1.00 (reference)	1.00 (reference)	
Stratified analysis by hs-CRP						
	Lower tertile					-
		Poor (n=75)	5.05 (1.75, 14.53)	3.87 (1.32, 11.40)	3.46 (1.14, 10.45)	
		Intermediate (n=472)	1.34 (0.57, 3.15)	1.06 (0.46, 2.45)	1.01 (0.44, 2.32)	
		Ideal (n=321)	1.00 (reference)	1.00 (reference)	1.00 (reference)	
	Middle tertile					-
		Poor (n=109)	2.76 (0.97, 7.83)	1.82 (0.62, 5.33)	2.79 (0.84, 9.20)	
		Intermediate (n=515)	0.64 (0.27, 1.49)	0.50 (0.21, 1.15)	0.57 (0.24, 1.35)	
		Ideal (n=253)	1.00 (reference)	1.00 (reference)	1.00 (reference)	
	Higher tertile					-
		Poor (n=158)	1.32 (0.45, 3.89)	0.91 (0.31, 2.67)	1.21 (0.39, 3.78)	
		Intermediate (n=533)	0.97 (0.39, 2.24)	0.74 (0.31, 1.75)	0.84 (0.35, 2.02)	
		Ideal (n=186)	1.00 (reference)	1.00 (reference)	1.00 (reference)	

Values are presented as odds ratio (95% confidence interval). MMSE-DS, Mini-Mental State Examination-Dementia Screening; SES, socioeconomic status; SBP, systolic blood pressure; hs-CRP, high-sensitivity C-reactive protein.

1 The CANHEART health index and its criteria defined by the Cardiovascular Health in Ambulatory Care Research Team in 2014.

2 Results from a logistic regression model with the penalized likelihood option; model 1: age and sex-adjusted model+adjustment for household income, education level, marital status, and drinking status; model 2: model 1+adjustment for smoking status, total cholesterol, fasting glucose, and SBP.

**Table 3.** Associations between the CANHEART health index and cognitive function in men and women by hs-CRP tertiles

CANHEART health index^1^				Low cognitive function (MMSE-DS<24)^2^			P for trend
				Age-adjusted model	Model 1, additionally adjusted for SES and drinking status	Model 2, additionally adjusted for health status	
Men (n=830)							
	Total						0.037
		Poor (n=274)		8.25 (0.47, 145.40)	7.07 (0.56, 88.93)	9.33 (0.76, 14.13)	
		Intermediate (n=279)		4.28 (0.25, 72.61)	4.62 (0.38, 55.90)	5.61 (0.49, 64.87)	
		Ideal (n=277)		1.00 (reference)	1.00 (reference)	1.00 (reference)	
	Stratified analysis by hs-CRP						
		Lower tertile					-
			Poor (n=41)	11.68 (0.64, 212.35)	9.06 (0.65, 126.07)	14.59 (0.94, 27.17)	
			Intermediate (n=168)	1.95 (0.10, 37.46)	3.86 (0.27, 55.28)	5.40 (0.36, 80.16)	
			Ideal (n=65)	1.00 (reference)	1.00 (reference)	1.00 (reference)	
		Middle tertile					-
			Poor (n=65)	0.50 (0.01, 23.13)	0.58 (0.03, 10.43)	0.83 (0.09, 7.39)	
			Intermediate (n=166)	1.30 (0.07, 24.86)	1.74 (0.19, 16.00)	1.28 (0.22, 7.39)	
			Ideal (n=48)	1.00 (reference)	1.00 (reference)	1.00 (reference)	
		Higher tertile					-
			Poor (n=78)	1.71 (0.08, 35.79)	1.15 (0.08, 17.34)	1.04 (0.08, 14.36)	
			Intermediate (n=171)	1.12 (0.06, 21.60)	0.88 (0.06, 12.32)	0.86 (0.07, 10.55)	
			Ideal (n=28)	1.00 (reference)	1.00 (reference)	1.00 (reference)	
Women (n=1,792)							
	Total						0.485
		Poor (n=158)		2.37 (1.27, 4.45)	1.61 (0.84, 3.09)	2.03 (1.00, 4.11)	
		Intermediate (n=1,015)		0.96 (0.58, 1.59)	0.76 (0.45, 1.28)	0.83 (0.49, 1.41)	
		Ideal (n=619)		1.00 (reference)	1.00 (reference)	1.00 (reference)	
	Stratified analysis by hs-CRP						
		Lower tertile					-
			Poor (n=24)	3.55 (1.03, 12.27)	2.81 (0.79, 9.96)	2.27 (0.62, 8.37)	
			Intermediate (n=304)	1.26 (0.52, 3.02)	1.00 (0.43, 2.37)	0.93 (0.39, 2.20)	
			Ideal (n=256)	1.00 (reference)	1.00 (reference)	1.00 (reference)	
		Middle tertile					-
			Poor (n=44)	3.36 (1.16, 9.67)	2.17 (0.70, 6.67)	3.44 (0.93, 12.73)	
			Intermediate (n=349)	0.55 (0.22, 1.34)	0.38 (0.15, 0.95)	0.43 (0.17, 1.10)	
			Ideal (n=205)	1.00 (reference)	1.00 (reference)	1.00 (reference)	
		Higher tertile					-
			Poor (n=80)	1.15 (0.35, 3.82)	0.81 (0.25, 2.69)	1.16 (0.32, 4.17)	
			Intermediate (n=362)	0.87 (0.35, 2.13)	0.68 (0.28, 1.68)	0.81 (0.32, 2.04)	
			Ideal (n=158)	1.00 (reference)	1.00 (reference)	1.00 (reference)	

Values are presented as odds ratio (95% confidence interval). MMSE-DS, Mini-Mental State Examination-Dementia Screening; SES, socioeconomic status; SBP, systolic blood pressure; hs-CRP, high-sensitivity C-reactive protein.

1 The CANHEART health index and its criteria defined by the Cardiovascular Health in Ambulatory Care Research Team in 2014.

2 Results from a logistic regression model with the penalized likelihood option; model 1: age-adjusted model+adjustment for household income, education level, marital status, and drinking status; model 2: model 1+adjustment for total cholesterol, fasting glucose, and SBP.

**Table 4.** Associations between each CANHEART health index component and cognitive function in men and women by hs-CRP tertiles (n=830)

CANHEART health index (each components)^1^			Cognitive dysfunction (MMSE-DS<24)^2^			
			Total	hs-CRP tertile		
				Lower	Middle	Higher
Men						
	Smoking					
		Current smoker (n=233)	0.70 (0.23, 2.09)	1.01 (0.19, 0.30)	0.65 (0.14, 3.01)	0.59 (0.14, 2.41)
		Non-smoker or former (n=597)	1.00 (reference)	1.00 (reference)	1.00 (reference)	1.00 (reference)
	Overweight, obesity (kg/m^2^)					
		BMI≥25 (n=366)	1.31 (0.49, 0.49)	3.37 (0.80, 14.26)	1.00 (0.25, 3.97)	1.27 (0.34, 4.78)
		BMI<25 (n=464)	1.00 (reference)	1.00 (reference)	1.00 (reference)	1.00 (reference)
	Leisure physical activity (min/d)					
		<30 (n=218)	1.27 (0.45, 3.57)	0.52 (0.09, 3.02)	0.73 (0.15, 3.62)	2.47 (0.68, 9.04)
		≥30 (n=612)	1.00 (reference)	1.00 (reference)	1.00 (reference)	1.00 (reference)
	Fruit and vegetable consumption (servings/d)					
		<5 (n=241)	2.04 (0.75, 5.53)	0.96 (0.21, 4.47)	1.49 (0.37, 5.97)	3.83 (0.91, 16.13)
		≥5 (n=589)	1.00 (reference)	1.00 (reference)	1.00 (reference)	1.00 (reference)
	Hypertension^3^					
		Yes (n=240)	3.79 (1.29, 11.10)	7.68 (1.66, 35.59)	0.88 (0.18, 4.30)	1.37 (0.35, 5.34)
		No (n=590)	1.00 (reference)	1.00 (reference)	1.00 (reference)	1.00 (reference)
	Diabetes^3^					
		Yes (n=87)	1.73 (0.37, 8.23)	4.81 (0.47, 49.71)	4.40 (0.80, 24.15)	0.55 (0.04, 8.33)
		No (n=743)	1.00 (reference)	1.00 (reference)	1.00 (reference)	1.00 (reference)
Women						
	Smoking					
		Current smoker (n=26)	0.25 (0.02, 4.27)	0.92 (0.04, 23.50)	1.51 (0.08, 29.72)	0.26 (0.01, 5.99)
		Non-smoker or former (n=1,766)	1.00 (reference)	1.00 (reference)	1.00 (reference)	1.00 (reference)
	Overweight, obesity (kg/m^2^)					
		BMI≥25 (n=520)	1.28 (0.78, 2.11)	2.05 (0.86, 4.88)	0.96 (0.41, 2.24)	1.25 (0.56, 2.81)
		BMI<25 (n=1,272)	1.00 (reference)	1.00 (reference)	1.00 (reference)	1.00 (reference)
	Leisure physical activity (min/d)					
		<30 (n=487)	1.02 (0.60, 1.73)	0.98 (0.40, 2.36)	1.03 (0.40, 2.65)	1.30 (0.55, 3.06)
		≥30 (n=1,305)	1.00 (reference)	1.00 (reference)	1.00 (reference)	1.00 (reference)
	Fruit and vegetable consumption (servings/d)					
		<5 (n=370)	1.44 (0.85, 2.44)	1.43 (0.58, 3.52)	2.52 (1.06, 6.00)	0.89 (0.35, 2.28)
		≥5 (n=1,422)	1.00 (reference)	1.00 (reference)	1.00 (reference)	1.00 (reference)
	Hypertension^3^					
		Yes (n=364)	0.93 (0.52, 1.65)	1.37 (0.54, 3.49)	0.65 (0.22, 1.92)	0.84 (0.34, 2.10)
		No (n=1,428)	1.00 (reference)	1.00 (reference)	1.00 (reference)	1.00 (reference)
	Diabetes^3^					
		Yes (n=97)	1.40 (0.51, 3.86)	0.43 (0.05, 4.05)	4.12 (0.80, 21.12)	1.32 (0.27, 6.53)
		No (n=1,695)	1.00 (reference)	1.00 (reference)	1.00 (reference)	1.00 (reference)

Values are presented as odds ratio (95% confidence interval). MMSE-DS, Mini-Mental State Examination-Dementia Screening; BMI, body mass index; OR, odds ratio; CI, confidence interval; SBP, systolic blood pressure; hs-CRP, high-sensitivity C-reactive protein.

1 The CANHEART health index and its criteria defined by the Cardiovascular Health in Ambulatory Care Research Team in 2014.

2 Results from a logistic regression model with the penalized likelihood option, adjusted for age, education level, household income, marital status, current drinking status, total cholesterol, fasting glucose level, and mean SBP.

3 Self-reported disease history (lifetime).

## SUPPLEMENTARY MATERIALS

Supplementary Material 1. Cardiovascular health index and ideal group criteria included components.

Supplementary Material 2. Association between Life’s Simple 7 score and cognitive function by gender (Men=830, Women=1,792)

Supplementary Material 3. Descriptive characteristics of participants included and excluded in sensitivity analyses.

Supplementary Material 4. Association between CANHEART health index and cognitive function by gender and hs CRP tertile (Men=727, Women=1,514)
