# Supplementary material for: Exploring the associations between cardiovascular health measured with the CANHEART model and early cognitive impairment in a middle-aged population in Korea
Source: Epidemiol Health. 2021 Jul 13;43:e2021044. doi: 10.4178/epih.e2021044 (PMC8510829; doi:10.4178/epih.e2021044)
Supplement: Supplementary Material 1. — Cardiovascular health index and ideal group criteria included components. [file epih-43-e2021044-suppl.docx]

**Supplementary** **Material 1**. Cardiovascular health index and ideal group criteria included components.

|  | **CANHEART health index** | **Life’s Simple 7 tools^1^** |
| --- | --- | --- |
| (1) Smoking | Never or former smoker (>1 year) | Never or former smoker (>1 year) |
| (2) Physical Activity | At least 150 min/week moderate intensit-y or 75 min/week vigorous intensity | At least 150 min/week moderate intensit-y or 75 min/week vigorous intensity |
| (3) Dietary Intake | Fruit and vegetables consumed ≥ 5 times/day | At least 1 servings of fruits and vegetables/day and 2 servings of fish/week |
| (4) Body Mass Index | < 25 kg/m^2^ | < 25 kg/m^2^ |
| (5) Total Cholesterol^2^ | - | Untreated and <200 mg/dL |
| (6) Blood Pressure | Self-reported hypertension diagnosed by a health care professional | Untreated and <80 mmHg |
| (7) Plasma Glucose | Self-reported diabetes diagnosed by a health care professional | Untreated and <100 mg/dL |

^1^ Life’s Simple 7 tools, which emphasized by American Heart Association (2010) and modified by Samieri C (2018), were used in this study.

^2^ Total cholesterol component not included in CANHEART health index by Cardiovascular Health in Ambulatory Care Research Team (2013)

**Supplementary Material 2**. Association between Life’s Simple 7 score and cognitive function by gender (Men=830, Women=1,792)

| Life’s Simple 7 tools ^1^ | **Low cognitive function (MMSE-DS < 24)** | | | | | | | | | | | | | |  |  |  |
| --- | --- | --- | --- | --- | --- | --- | --- | --- | --- | --- | --- | --- | --- | --- | --- | --- | --- |
|  | Age adjusted model | | | | | Model 1, additionally adjusted for SES and drinking status | | | | | Model 2, additionally adjusted for health status | | | |  |  |  |
|  | OR^2^ | | (95% CI) | | OR^2^ | | | (95% CI) | | OR^2^ | | | (95% CI) | |  |  |  |
| **Total (N=2,622)** |  |  | |  | |  |  | |  | |  |  | |  |  |  |  |
| Poor (n=461) | 1.49 | (0.89 – 2.75) | | | | 1.08 | (0.58 – 1.99) | | | | 1.38 | (0.69 – 2.77) | | |  |  |  |
| Intermediate (n=1,329) | 1.01 | (0.36 – 1.66) | | | | 0.91 | (0.56 – 1.50) | | | | 0.98 | (0.58 – 1.65) | | |  |  |  |
| Ideal (n=832) | 1.00 | ref | |  | | 1.00 | ref | |  | | 1.00 | ref | |  |  |  |  |
| **Total men (N=830)** |  |  | |  | |  |  | |  | |  |  | |  |  |  |  |
| Poor (n=231) | 1.27 | (0.27 - 5.97) | | | | 1.27 | (0.30 - 5.30) | | | | 1.71 | (0.37 - 7.93) | | |  |  |  |
| Intermediate (n=425) | 1.04 | (0.24 - 4.45) | | | | 1.00 | (0.26 - 3.87) | | | | 1.43 | (0.35 - 5.79) | | |  |  |  |
| Ideal (n=174) | 1.00 | ref | | | | 1.00 | ref | | | | 1.00 | ref | | |  |  |  |
| **Total women (N=1,792)** |  |  | | | |  |  | | | |  |  | | |  |  |  |
| Poor (n=230) | 1.45 | (0.74 - 2.82) | | | | 1.05 | (0.53 - 2.06) | | | | 1.20 | (0.55 - 2.58) | | |  |  |  |
| Intermediate (n=904) | 0.97 | (0.25 - 1.64) | | | | 0.86 | (0.51 - 1.45) | | | | 0.90 | (0.51 – 1.56) | | |  |  |  |
| Ideal (n=658) | 1.00 | ref | | | | 1.00 | ref | | | | 1.00 | ref | | |  |  |  |

^1^ Life’s Simple 7 score emphasized by American Heart Association in 2010

^2^ Results from logistic regression model with penalized likelihood option; Model 1: Age adjusted model + house income, education level, marital status, and drinking status adjusted; Model 2: Model 1 + Total cholesterol, fasting glucose, and SBP adjusted

**Abbreviation**: MMSE-DS, Mini-Mental State Examination-Dementia Screening; SES: socio-economic status; OR, Odds Ratio; CI: Confidence interval; SBP, Systolic Blood Pressure; DBP; hs CRP: high-sensitivity C-reactive protein

**Supplementary Material 3**. Descriptive characteristics of participants included and excluded in sensitivity analyses.

| Variables | Participants included | |  | Participants excluded | | *P* value |
| --- | --- | --- | --- | --- | --- | --- |
|  | in sensitivity analyses | |  | in sensitivity analyses | |  |
|  | (n=2,241) | |  | (n=381) | |  |
| Age, years, mean ± SD | 57.4 | ±3.9 |  | 56.5 | ±3.6 | <.001 |
| Gender, N(%) |  |  |  |  |  |  |
| Men | 727 | (32.4) |  | 103 | (27.0) | 0.036 |
| Women | 1,514 | (67.6) |  | 75 | (73.0) |  |
| Education attainment years, N(%) |  |  |  |  |  |  |
| ≤6 years | 169 | (7.5) |  | 49 | (12.9) | <.001 |
| 6-9 years | 262 | (11.7) |  | 64 | (16.8) |  |
| 9-12 years | 953 | (42.5) |  | 165 | (43.3) |  |
| 12+ years | 857 | (38.3) |  | 103 | (27.0) |  |
| Family income(year), N(%) |  |  |  |  |  |  |
| Q1 | 517 | (23.1) |  | 136 | (35.7) | <.001 |
| Q2 | 670 | (29.9) |  | 102 | (26.8) |  |
| Q3 | 421 | (18.8) |  | 89 | (23.4) |  |
| Q4 | 633 | (28.3) |  | 54 | (14.2) |  |
| Marital status, N(%) |  |  |  |  |  |  |
| Unmarried | 25 | (1.1) |  | 2 | (0.5) | 0.714 |
| Married-death of spouse | 127 | (5.7) |  | 24 | (6.3) |  |
| Married-separated | 124 | (5.5) |  | 22 | (5.8) |  |
| Married-living together | 1,965 | (87.7) |  | 333 | (87.4) |  |
| Smoking, N(%) |  |  |  |  |  |  |
| Non-smoker | 1,616 | (72.1) |  | 293 | (76.9) | 0.038 |
| Former smoker | 429 | (19.1) |  | 52 | (13.7) |  |
| Current smoker | 196 | (8.9) |  | 36 | (9.5) |  |
| Drinking, N(%) |  |  |  |  |  |  |
| Non-drinker | 509 | (22.7) |  | 161 | (42.3) | <.001 |
| Former drinker | 108 | (4.8) |  | 18 | (4.7) |  |
| Current drinker | 1,624 | (72.5) |  | 202 | (53.0) |  |
| Regular physical activity, N(%)^1^ |  |  |  |  |  |  |
| No | 573 | (25.6) |  | 132 | (34.7) | <.001 |
| Yes | 1,668 | (74.4) |  | 249 | (65.4) |  |
| Body mass index, mean ± SD | 24.0 | ±2.9 |  | 23.9 | ±2.8 | 0.487 |
| Ever had hypertension, N(%)^2^ |  |  |  |  |  |  |
| No | 1,718 | (76.7) |  | 300 | (78.7) | 0.373 |
| Yes | 523 | (23.3) |  | 281 | (21.3) |  |
| SBP, mmHg, mean ± SD | 120.7 | ±15.3 |  | 118.0 | ±13.7 | <.001 |
| DBP, mmHg, mean ± SD | 76.8 | ±9.8 |  | 75.5 | ±8.6 | 0.008 |
| Ever had DM, N(%)^2^ |  |  |  |  |  |  |
| No | 2,085 | (93.0) |  | 353 | (92.7) | 0.784 |
| Yes | 156 | (7.0) |  | 28 | (7.4) |  |
| Fasting insulin, uIU/mL, mean ± SD | 8.9 | ±3.7 |  | 8.5 | ±3.6 | 0.03 |
| Fasting glucose, mg/dL, mean ± SD | 93.8 | ±19.5 |  | 94.3 | ±24.4 | 0.678 |
| HbA1c, %, mean ± SD | 5.8 | ±0.7 |  | 5.8 | ±0.8 | 0.762 |
| hs CRP, mg/L, mean ± SD | 1.4 | ±3.4 |  | 1.7 | ±4.7 | 0.323 |
| CANHEART health index, N(%)^3^ |  |  |  |  |  |  |
| Poor | 282 | (12.6) |  | 60 | (15.8) | 0.238 |
| Intermediate | 1,306 | (58.3) |  | 214 | (56.2) |  |
| Ideal | 653 | (29.1) |  | 107 | (28.1) |  |

^1^ Participants walking at least 30 minutes per day grouped into regular physical activity group

^2^ Self-reported disease history

^3^ CANHEART health index and its criteria emphasized by Cardiovascular Health in Ambulatory Care Research Team in 2014

**Abbreviation**: MMSE-DS, Mini-Mental State Examination-Dementia Screening; SD, Standard Deviation; SBP, Systolic Blood Pressure; DBP, Diastolic Blood Pressure; DM: Diabetes mellitus; HbA1c: hemoglobin A1c; hs CRP: high-sensitivity C-reactive protein

**Supplementary Material 4**. Association between CANHEART health index and cognitive function by gender and hs CRP tertile (Men=727, Women=1,514)

| CANHEART health index ^1^ | **Low cognitive function (MMSE-DS < 24)** | | | | | | | | | | | | | |  |  |  |
| --- | --- | --- | --- | --- | --- | --- | --- | --- | --- | --- | --- | --- | --- | --- | --- | --- | --- |
|  | hs-CRP, lower tertile | | | | | hs-CRP, middle tertile | | | | | hs-CRP, higher tertile | | | |  |  |  |
|  | OR^2^ | | (95% CI) | | OR^2^ | | | (95% CI) | | OR^2^ | | | (95% CI) | |  |  |  |
| **Total (N=2,241)** |  |  | |  | |  |  | |  | |  |  | |  |  |  |  |
| Poor (n=282) | 4.65 | (1.36 – 15.92) | | | | 2.55 | (0.63 – 10.31) | | | | 1.25 | (0.29 – 5.44) | | |  |  |  |
| Intermediate (n=1,306) | 0.93 | (0.36 – 2.41) | | | | 0.38 | (0.14 – 1.01) | | | | 1.19 | (0.41 – 3.45) | | |  |  |  |
| Ideal (n=653) | 1.00 | ref | |  | | 1.00 | ref | |  | | 1.00 | ref | |  |  |  |  |
| **Total men (N=727)** |  |  | |  | |  |  | |  | |  |  | |  |  |  |  |
| Poor (n=163) | 6.74 | (0.84 - 54.36) | | | | N/A | N/A | | | | 0.94 | (0.06 - 13.95) | | |  |  |  |
| Intermediate (n=445) | 0.93 | (0.11 - 7.67) | | | | N/A | N/A | | | | 0.48 | (0.03 – 6.81) | | |  |  |  |
| Ideal (n=119) | 1.00 | ref | | | | 1.00 | ref | | | | 1.00 | ref | | |  |  |  |
| **Total women (N=1,514)** |  |  | | | |  |  | | | |  |  | | |  |  |  |
| Poor (n=119) | 2.23 | (0.51 - 9.70) | | | | 2.87 | (0.63 - 13.18) | | | | 0.93 | (0.14 - 6.22) | | |  |  |  |
| Intermediate (n=861) | 0.96 | (0.37 - 2.49) | | | | 0.29 | (0.10 - 0.85) | | | | 1.47 | (0.48 – 4.48) | | |  |  |  |
| Ideal (n=534) | 1.00 | ref | | | | 1.00 | ref | | | | 1.00 | ref | | |  |  |  |

^1^ CANHEART health index and its criteria emphasized by Cardiovascular Health in Ambulatory Care Research Team in 2014

^2^ Results from logistic regression model with penalized likelihood option; Adjusted for age, house income, education level, marital status, drinking status, total cholesterol, fasting glucose, and SBP.

**Abbreviation**: MMSE-DS, Mini-Mental State Examination-Dementia Screening; OR, Odds Ratio; CI: Confidence interval; SBP, Systolic Blood Pressure; DBP; hs CRP: high-sensitivity C-reactive protein
